# *Klebsiella aerogenes* ST117 causing folliculitis in men having sex with men, Belgium, February 2025

**DOI:** 10.2807/1560-7917.ES.2025.30.20.2500304

**Published:** 2025-05-22

**Authors:** Nicolas Yin, Jonathan Krygier, Célestin Mairesse, Agnès Libois, Sophie Quoilin, Delphine Martiny

**Affiliations:** 1 Department of Microbiology, Laboratoire Hospitalier Universitaire de Bruxelles – Universitair Laboratorium Brussel (LHUB-ULB), Université Libre de Bruxelles, Brussels, Belgium; 2 Department of Dermatology, CHU Saint-Pierre, Université Libre de Bruxelles, Brussels, Belgium; 3 Department of Infectious Diseases, CHU Saint-Pierre, Université Libre de Bruxelles, Brussels, Belgium; 4 Infection Control and Prevention Unit, CHU Saint-Pierre, Université Libre de Bruxelles, Brussels, Belgium; 5 Faculty of Medicine and Pharmacy, University of Mons (UMONS), Mons, Belgium

**Keywords:** *Klebsiella aerogenes*, Folliculitis, Men Who Have Sex With Men, Belgium

## Abstract

*Klebsiella aerogenes* has recently been reported as a causative agent of folliculitis in men who have sex with men (MSM). We present four cases of folliculitis in MSM diagnosed in Brussels, Belgium. Patients were aged between 25 and 50 years, and all were infected by a single multilocus sequence type (ST117) strain. This strain carried the yersiniabactin siderophore genes. Following a preliminary treatment by sulfamethoxazole−trimethoprim for 7−14 days, all patients experienced a recurrence of symptoms, necessitating an extended therapeutic regimen.

*Klebsiella aerogenes* (formerly *Enterobacter aerogenes*) is rarely reported as a cause of skin and soft tissue infection (SSTI) [1]. However, it has recently been described as a causal agent for folliculitis, particularly among men having sex with men (MSM) [2]. Here, we report four patients with folliculitis caused by *K. aerogenes* in February 2025, in Brussels, Belgium.

## Description of the cases

**Figure 1:**
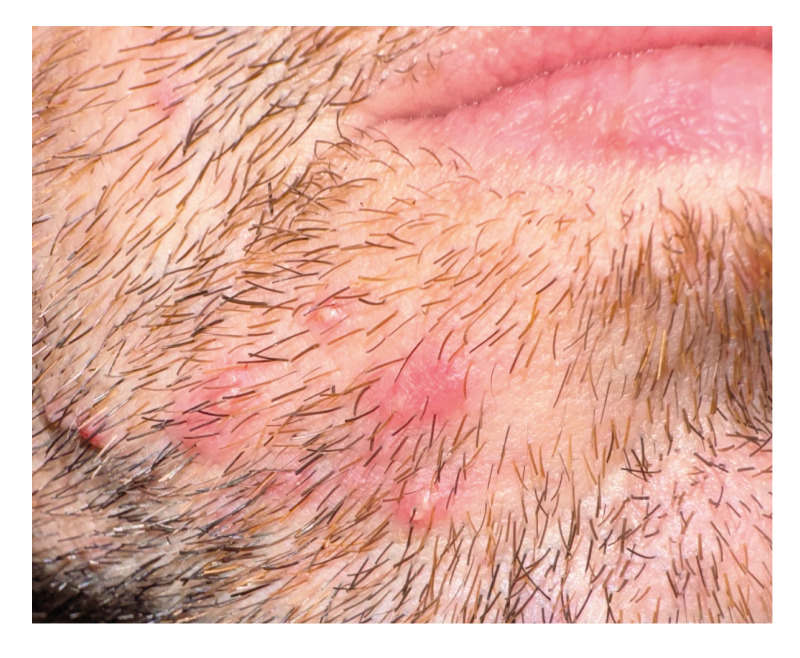
Folliculitis caused by *Klebsiella aerogenes* ST117 in men having sex with men, Brussels, Belgium, February 2025

In February 2025, four patients exhibiting clinical signs of folliculitis attended the dermatology clinic of the infectious disease department of a hospital in Brussels, Belgium. This clinic is specialised in the treatment of sexually transmitted infections. All the patients presented with erythematous follicular papules and pustules, some of which developed into painful nodules. These were located on the beard area, cheeks, chin, and occasionally the frontal scalp (Figure 1).

ST: sequence type.

Lesions are characterised by multiple erythematous follicular papules and pustules, some coalescing into painful nodules.

The patients were all male, aged between 25 and 50 years, and reported having sex with men (Table). Three patients each had been diagnosed 7 to 15 months earlier with folliculitis, which since had either persisted or recurred several times. Only one of them had *K. aerogenes* identified on a pustule swab 2 months earlier, but multilocus sequence typing was not performed at that time. Visiting community hot tubs for leisure and meeting sexual partners, which was prior described for cases of folliculitis in France [2]*,* was only reported by one of the four patients, with this visit having occurred within the previous month. They did not report travelling abroad, but they were not asked about short-distance travel, such as travelling to Paris (about 300 km away). Two had a regular partner, but neither of these partners exhibited clinical signs of folliculitis. None of the patients were aware of any similar case among their relatives or sexual partners. None of them went to a barber to have their beards cared for. No other epidemiological links were found between the cases.

**Table:** Characteristics of patients with *Klebsiella aerogenes* ST117 folliculitis, Brussels, Belgium, February 2025 (n = 4 cases)

Case nr	Age group in years	MSM	Living with HIV	Antibiotic resistance	Regular partner	Evolution since	Previous treatments^a^	Treatment	Follow-up
1	25–50	Yes	No	None	Yes^b^	NA	NA	Sulfamethoxazole−trimethoprim bid 7 days	Recurrence 10 days post-treatment
2	25–50	Yes	No	None	Yes^b^	Jul 2024	Flucloxacillin, doxycycline, amoxicillin–clavulanic acid, clindamycin	Sulfamethoxazole−trimethoprim bid 7 days	Recurrence 3 weeks post-treatment
3	25–50	Yes	No	None	No	Jan 2024	Ciprofloxacin	Sulfamethoxazole−trimethoprim bid 10 days	Recurrence 1 month post-treatment
4	25–50	Yes	No	Ciprofloxacine, piperacillin–tazobactam, third-generation cephalosporin	No	Oct 2022	Ciprofloxacin, tetracycline	Sulfamethoxazole−trimethoprim bid 14 days	Recurrence 10 days post-treatment

Bid: bis in die; MSM: man reporting having sex with men; NA: not applicable; nr: number; ST: sequence type.

^a^ Patients who received previous treatments had received these in other clinics in Belgium than the one concerned by the current report.

^b^ No folliculitis was observed in the partner.

At the hospital in Brussels, lesions from the four patients were swabbed and samples were sent to the clinical laboratory for aerobic culture as part of standard care protocol. Cultures of pustule swabs of all patients were positive for the *K. aerogenes* species, which was identified by Matrix-assisted laser desorption ionisation-time of flight (MALDI-TOF) mass spectrometry (Sirius, MBT IVD reference library version 2023, 22 Bruker Daltonics, Bremen, Germany). Antimicrobial susceptibility testing using the VITEK 2 AST-N446 assay (Biomérieux, Marcy-l'Étoile, France) revealed no acquired resistance to β-lactams, ciprofloxacin, or sulfamethoxazole−trimethoprim in three of them. The strain cultured from the fourth patient exhibited resistance to ciprofloxacin and to third-generation cephalosporins and piperacillin–tazobactam via a cephalosporinase de-repression mechanism.

Patients were treated with sulfamethoxazole−trimethoprim 800/160 mg bis in die (bid) for 7–14 days. However, all patients relapsed between 10 days and 1 month after completing the initial course of sulfamethoxazole−trimethoprim treatment, requiring an additional prolonged course of therapy with sulfamethoxazole−trimethoprim for at least 3 weeks. At the time of writing this article, one of the four patients had a recurrence. On this occasion, carriage sampling was performed and his partner tested positive for *K. aerogenes* in a nasal swab. A new course of sulfamethoxazole−trimethoprim was started, as well as decontamination of the partner with topical tobramycin. No recurrence has been reported for others, but surveillance is ongoing.

## Molecular typing and phylogenetic investigations

The *K. aerogenes* strains were subjected to whole genome sequencing (WGS). The extraction of DNA was performed using the EZ1 and 2 Virus Mini Kit v2.0 (Qiagen, Hilden, Germany) and the EZ2 Connect MDx instrument (Qiagen). Genomic DNA was enzymatically fragmented and modified to generate an Illumina-compatible DNA library using Revelo DNA-Seq for MagicPrep NGS (Tecan, Männedorf, Switzerland). The library was sequenced using a MiniSeq machine (Illumina Inc., San Diego, United State (US)) with MiniSeq Mid Output Kit (300 cycles) in 2 × 150 bp (bp) pair-end mode. The de novo assembly was performed using the Velvet algorithm (version 1.1.04) on Ridom SeqSphere + version 10.0.5 (Ridom GmbH, Münster, Germany).

The *K. aerogenes* multilocus sequence type (MLST) was determined using Ridom SeqSphere. The WGS single nt polymorphism (wgSNP) analysis was performed using Bionumerics software version 8.1 (Biomérieux, Marcy-l'Étoile, France) using the genome of a *K. aerogenes* isolated from MSM in China with an identical sequence type (ST) (GenBank accession GCA_030133885.1) as a mapping reference [3]. Potential virulence factors were researched in the Virulence Factor Database (VFDB) for the *Klebsiella* genus [4].

**Figure 2:**
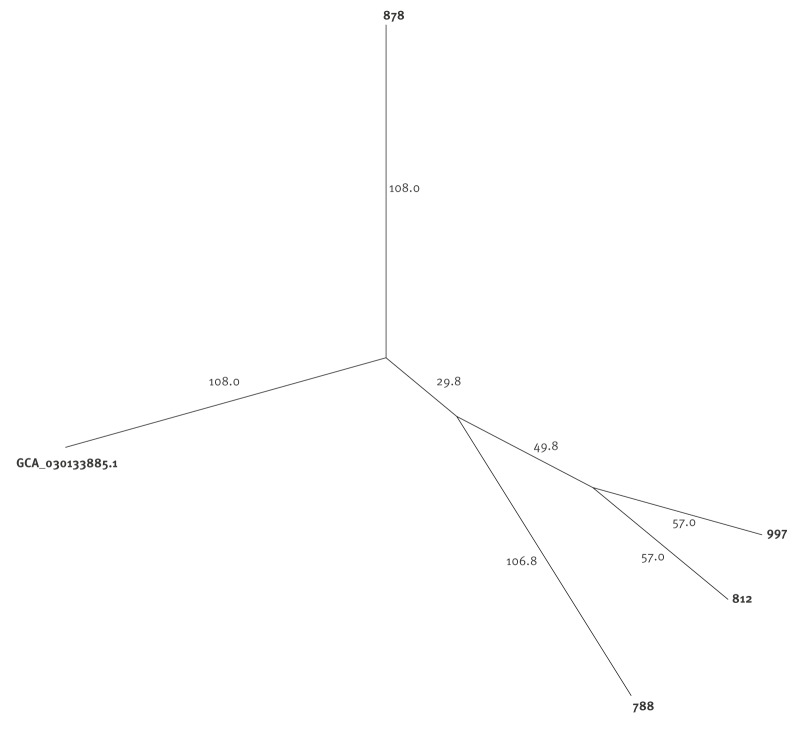
Phylogenetic unrooted tree generated from whole-genome sequencing of *Klebsiella aerogenes* isolated from folliculitis, Brussels, Belgium, February 2025 (n = 5 whole genome sequences)

The MLST analysis revealed that all the strains belonged all to ST117. The wgSNP analysis showed a distance between 57 to 148 SNPs between the clinical strains (Figure 2). Interestingly, one clinical strain had a lesser SNP distance (108 SNPs) with the reference used for mapping than with the other clinical strains (141–152 SNPs). Using VFDB showed that all strains carried the yersiniabactin siderophores genes. Only one carried colibactin genes. However, yersiniabactin siderophores genes were also present in the *K. aerogenes* ST117 used as a reference (GenBank accession number: GCA_030133885.1). The detailed results of VFDB are available in Supplementary Table S1.

The tree was generated using the categorical distance algorithm for single nucleotide polymorphisms (SNPs) and the unweighted pair group method with arithmetic mean (UPGMA) on Bionumerics 8.1 software (strain numbers are underlined). The sequence with GenBank accession GCA_030133885.1 was used as a mapping reference. The other sequences which were used for the phylogenetic analysis and derived from the four patients’ respective isolates are in bold font.

## Discussion

We report here four cases of folliculitis caused by *K. aerogenes* affecting MSM in Belgium during the first quarter of 2025. All of them were caused by a specific clone of *K. aerogenes* belonging to ST117, exhibiting no more than 152 SNPs between the clinical strains. A study conducted in Guangzhou, China, in 2019 examined *K. aerogenes* colonising the nasal cavities of 258 MSM. The most prevalent STs were ST4, ST93, and ST14, while ST117 was detected in only two isolates [3]. Consequently, *K. aerogenes* ST117 may be especially adapted to cause recurring folliculitis. Virulence factors specific to *K. aerogenes* are not well known. Yersiniabactin and colibactin have been proposed as potential virulence factors for *K. aerogenes* and *K. aerogenes* genomes could be clustered based on the presence/absence of these virulence factors [4,5].

Our laboratory performs microbiological analysis for inpatients and outpatients of two general hospitals, as well as for patients attending their sampling centres in the Brussels region, Belgium, covering a service area of 700,000 inhabitants. In the initial quarter of 2025, *K. aerogenes* was isolated from pustule swabs from folliculitis only from five patients in our laboratory, including the four cases reported here. The fifth patient was also an MSM attending the same dermatology clinic, but the strain of this patient was discarded before further analysis. We are not aware of other cases in Belgium and no new case has been identified via our laboratory in March and April 2025.

This infection appears to affect MSM to a greater extent than other demographics. In France, a recent report described seven male patients with facial folliculitis caused by *K. aerogenes*, who had consulted a dermatological practice or a hospital in the Paris area between 2016 and 2022. Among these patients, six were MSM. Therefore, it was hypothesised that this may be a new sexually transmitted infection [2]. In the French investigation, the strains of *K. aerogenes* were not typed so the potential involvement of a specific clone of *K. aerogenes* was not identified. In our investigation, regular partners of patients did not exhibit clinical signs, so occurrences might be also explained by individual susceptibilities to infection, which should be explored further. Such susceptibilities could possibly be due to behavioural and environmental (e.g. shaving methods, use of cosmetic products, hygiene), or immunological (type of immune response) factors. It is worth noting that none of the patients here were living with HIV or had an immunodeficiency that was known about. They nevertheless had frequent recurrence after initial clinical cure, as did the patients observed in France [2].

As they were deemed useful in SSTIs caused by other *Staphylococcus aureus* [6,7], the potential impact of prolonged antimicrobial treatment, the use of a decontamination protocol or additional hygienic measures should be assessed. Among aetiologies of SSTI, *K. aerogenes* is uncommon [1] and may be disregarded as a cause of folliculitis by clinicians, leading to potential treatment failure when using tetracyclines as a common empiric treatment since these antibiotics have limited effectiveness against *K. aerogenes* [8].

As previously reported in France [2], *K. aerogenes* folliculitis may also be emerging in Belgium in MSM and seems to be responsible for recurring infections. A single clone, *K. aerogenes* ST117 has been identified among four cases. 

This study has several limitations. It is limited to strains received in only one, although large, clinical laboratory in Belgium, Brussels. The bacterial strains in question are not typically stored in a strain library as they do not exhibit unusual antimicrobial resistance patterns or originate from precious sampling sites. Therefore, only a small number of strains could be analysed, and it was not possible to compare them to similar cases as no sequence have been published previously. Patients were not asked about travel in Europe and more particularly to France or Paris. However, the cities of Brussels and Paris (where previous cases were reported) are close to each other, situated approximately 300 km apart, and are connected by a high-throughput transportation network. Except for one patient and his partner, who were assessed for carriage upon the patient’s latest folliculitis reappearance, nasal or throat carriage of other patients and partners was not assessed and may be an explanation for recurrence.

## Conclusion

This report describes the diagnosis in Brussels, Belgium, of folliculitis caused by *Klebsiella aerogenes* ST117 in four patients self-reported as MSM. Following a preliminary treatment by sulfamethoxazole−trimethoprim for 7−14 days, symptoms recurred in all cases, necessitating an extended therapeutic regimen. With folliculitis caused by *Klebsiella aerogenes* having also recently been described in France*,* further investigations are needed to confirm if the ST177 clone is emerging in Europe and to determine its selective advantage in this pathology, as well as risk factors.

## Data Availability

The Whole Genome Shotgun project has been deposited at DDBJ/ENA/GenBank under the accession PRJNA1250536.

## Supplementary Data

62000 Supplementary Table S1
